# 类Richter´s综合征表现为ALK^+^间变大T细胞淋巴瘤一例报告并文献复习

**DOI:** 10.3760/cma.j.issn.0253-2727.2021.12.016

**Published:** 2021-12

**Authors:** 梦娟 李, 冰洁 丁, 柳 刘, 璐 王, 建平 刘, 佩佩 徐, 可树 周, 虎 周, 旭东 魏, 永平 宋

**Affiliations:** 河南省肿瘤医院，郑州大学附属肿瘤医院血液科，郑州 450008 Department of Hematology, Henan Cancer Hospital, The Affiliated Cancer Hospital of Zhengzhou University, Zhengzhou 450008, China

Richter's综合征（Richter's syndrome，RS）是慢性淋巴细胞白血病（CLL）/小淋巴细胞性淋巴瘤（SLL）向高级别非霍奇金淋巴瘤（NHL）或霍奇金淋巴瘤（HL）进展的统称，既往报道3％～5％的CLL/SLL患者会出现这种转化[1]–[2]。大多数RS为弥漫大B细胞淋巴瘤（DLBCL）。CLL/SLL进展为高侵袭性T细胞淋巴瘤者少见，进展为ALK^+^间变大T细胞淋巴瘤（anaplastic large T cell lymphoma, ALTCL）者罕有报道。我们收治了1例50岁男性患者，在诊断为CLL/SLL两年余后进展为ALK^+^ ALTCL，疾病快速进展直至死亡。现将该患者临床资料及治疗经过报道如下，并结合相关文献进行讨论。

## 病例资料

患者，男，50岁，因“发现颈部双侧淋巴结肿大1年余”于2018年3月27日入院。患者于2017年出现颈部双侧淋巴结肿大，于当地医院按淋巴结结核治疗后进行性增大。2018年2月颈部淋巴结病理活检示B细胞NHL。患者入院后血常规示淋巴细胞计数14.04×10^9^/L。增强CT示双侧颈根部、腋窝、锁骨上下、颈背部间隙内、纵隔内、双肺门、髂血管走行区、双侧腹股沟、盆腔、腹腔内、腹膜后、肝胃间多发淋巴结肿大，脾脏增大。骨髓细胞形态学示有核细胞增生活跃，淋巴细胞比例偏高（成熟淋巴细胞0.76）。白血病免疫分型示异常成熟B淋巴细胞约占有核细胞的0.54，表达CD19、CD5、CD23、CD200、CD43、κ，弱表达CD20、CD22、CD79b、CD25，细胞体积偏小，符合CLL表型（图1）。染色体：46，XY[20]。FISH检测CLL相关基因示：ATM（+），余阴性。患者明确诊断为CLL/SLL，Rai分期Ⅱ期，Binet分期B期。于2018年4月10日予患者RFC方案化疗，具体为：利妥昔单抗600 mg 每日1次，第0天；氟达拉滨40 mg每日1次，第1～3天；环磷酰胺400 mg，每日1次，第1～3天。每28 d为1个疗程，共化疗4个疗程，评价为完全缓解（CR）。患者2018年10月至2019年10月定期复查均提示CR。患者2020年9月上旬出现发热，最高体温39.2 °C，于2020年9月19日再次入院。查体示：咽红、双肺呼吸音粗，余无明显阳性体征。血常规、生化常规、凝血功能、甲状腺功能及抗核抗体谱均无异常；C反应蛋白140.78 mg/L，降钙素原0.094 µg/L；肺部影像学提示两肺炎性改变。骨髓细胞形态学检查及白血病微小残留病均未见异常成熟B淋巴细胞。多次血培养均阴性，结核相关检查阴性，多次真菌检查阴性。但患者反复发热，最高可达40 °C，且中性粒细胞计数逐渐增高至24.19×10^9^/L；铁蛋白440.00 µg/L；甘油三酯0.59 mmol/L；EB病毒（EBV）DNA 1.16×10^4^拷贝/ml，多次调整抗感染药物，发热仍未控制。2020年10月患者出现颈部双侧淋巴结肿大，脾区有压痛，影像学证实脾脏较前明显增大，甲丙线4 cm，且淋巴细胞计数增高，最高达10.52×10^9^/L（淋巴细胞百分比0.65）；余两系进行性下降。患者于2020年10月23日行左颈肿物切除活检，送检病理并行病原微生物相关检查。2020年10月26日复查骨髓细胞学示有核细胞增生活跃，成熟淋巴细胞0.548，易见网状组织细胞及噬血现象。白血病免疫分型示：Ⅰ门为异常成熟B淋巴细胞群，约占有核细胞的0.004，表达CD19、κ，弱表达CD22、CD45，细胞体积不大。CD3^−^CD4^+^门为异常成熟T淋巴细胞群，约占有核细胞的0.52，表达CD4、CD7、CD2、CD26、CD45、CD45RO、cCD3、CD38、HLA-DR，符合CD4^+^CD8^−^成熟T细胞淋巴瘤表型（图2）。（左颈淋巴结）病理诊断符合ALK阳性ALTCL。免疫组化CD3（−）、CD21（−）、Ki-67（80％+）、CD30（+）、CD4（+）、CD8（−）、CD5（−）、ALK（核质+）、GramB（+）、TIA-1（+）、EMA（+）、CD2（+）、CD7（+）（图3）；原位杂交结果：EBER阴性；BCR基因重排（+），TCR基因重排（+）。患者EBV-DNA转阴，铁蛋白升高至3991.00 µg/L，甘油三酯升高至2.38 mmol/L。患者体温>38 °C超过7 d、脾大、血细胞减少，累及外周血两系；血清铁蛋白及甘油三酯升高，根据2018版《噬血细胞综合征诊治中国专家共识》[3]，患者可诊断为噬血细胞综合征。患者明确诊断为ALK阳性ALTCL伴噬血细胞综合征。2020年10月28日起予西达本胺片30 mg 每周2次，联合HLH-2004方案治疗[4]，患者发热症状好转。11月17日患者骨髓有核细胞增生减低，成熟小淋巴细胞占0.655；外周血涂片中成熟小淋巴细胞占0.950。考虑疾病未缓解，继续治疗后出现重度骨髓抑制伴发热，后患者疾病持续进展，于2020年11月21日因多脏器功能衰竭死亡。

**图1:**
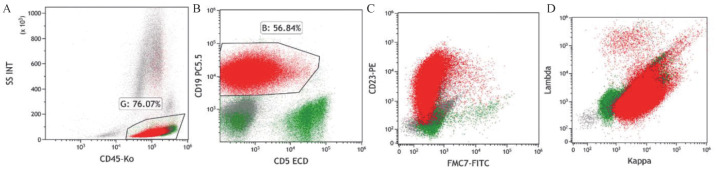
本例患者诊断慢性淋巴细胞白血病/小淋巴细胞性淋巴瘤时骨髓特征性免疫表型 A：以CD45/SSC设门分析成熟淋巴细胞群；B：CD5^+^CD19^+^异常淋巴细胞群；C：异常淋巴细胞FMC7^+^CD23^+^；D：κ限制性表达

**图2:**
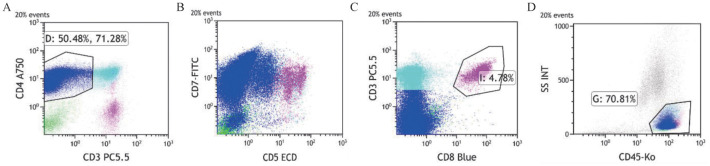
本例患者进展为ALK^+^间变大T细胞淋巴瘤时骨髓特征性免疫表型 A：CD3^−^CD4^+^异常淋巴细胞群；B：异常淋巴细胞CD5^−^CD7^+^；C；异常淋巴细胞CD3^−^CD8^−^：D：以CD45/SCC设门分析成熟淋巴细胞群

**图3:**
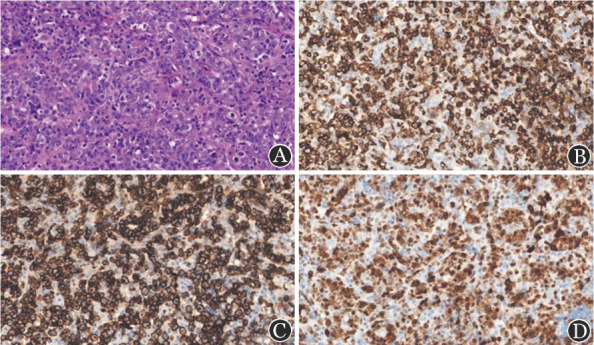
本例患者进展为ALK^+^间变大T细胞淋巴瘤时淋巴结活检HE染色及免疫组化染色结果（×400） A：HE染色；B：CD7染色；C：CD30染色；D：ALK染色

## 讨论及文献复习

RS是CLL/SLL转变为侵袭性淋巴瘤的特征，特点是症状突然加重，出现发热、体重减轻、淋巴结肿大及血清乳酸脱氢酶（LDH）水平升高[5]。这种现象1928年被Richter[6]首次报道，然而RS的定义则是Lortholary等[7]在1964年提出的，RS主要表现为DLBCL及HL，但随着越来越多关于CLL/SLL进展或合并T细胞谱系淋巴瘤的报道，确切的RS定义目前仍充满争议，有学者提出将这种非典型RS进展为T细胞谱系的淋巴瘤命名为“T rex”淋巴瘤[8]。

既往已有多篇文献描述了CLL/SLL进展为高级别T细胞谱系淋巴瘤的病例报道，其特征是疾病进展快，预后极差。Strickler等[9]报道了两例CLL/SLL患者经淋巴结活检证实CLL/SLL与T细胞淋巴瘤并存，作者认为其是一种非典型RS。Lee等[10]也认可Strickler等提出的概念，认为CLL/SLL后出现的EBV不相关的高度恶性T细胞淋巴瘤是RS的一种异常变体。已知EBV与许多淋巴瘤的发展有关，多达16％的RS可能存在EBV阳性，表明在免疫功能低下的宿主中潜在的EBV感染具有致病性[11]–[13]。Ansell等[11]在一例转化为侵袭性T细胞淋巴瘤患者的肿瘤性T细胞中发现了EBV，而在CLL/SLL细胞中EBV呈阴性，作者认为是EBV感染T细胞或成熟前干细胞导致T细胞淋巴瘤发生。该患者曾在外周血中检测到EBV阳性，尽管后期转阴，且淋巴结病理EBER阴性，但仍不排除其为疾病进展的始动因素。

T细胞淋巴瘤通常与慢性抗原刺激或慢性免疫抑制有关，有文献证明CLL/ SLL患者常伴随免疫监视减弱，细胞免疫研究发现其T细胞功能降低，CD8^+^T细胞克隆反常扩增且循环中CD8^+^T细胞计数增加[14]。CLL/SLL后T细胞淋巴瘤可以是辅助性T细胞型，也可以是细胞毒性T细胞表型。Novogrudsky等[15]报告了一例CLL/SLL进展为辅助性T细胞表型的高级别T细胞淋巴瘤，并称其为RS的异常表现。Martinez等[16]收集了6例具有细胞毒性T细胞表型的CLL/SLL进展的T细胞淋巴瘤，作者同样认为这种外周T细胞淋巴瘤应与典型的RS区分。与普通的T细胞淋巴瘤相比，已报道的CLL/SLL相关外周T细胞淋巴瘤非特指型病例均表达细胞毒性颗粒蛋白，在我们报道的病例中，ALTCL也表达细胞毒性蛋白TIA-1和颗粒酶B，表型提示为辅助性T细胞型。该患者曾行4个周期RFC方案治疗，CLL/SLL相关T细胞淋巴瘤的报道中很多患者既往未化疗，如文献[17]报道的下颌下腺复合肿瘤及文中回顾的3例患者均未接受任何治疗。且既往文献报道认为RFC方案并不增加第二肿瘤的发生率[18]–[19]。

该患者疾病进展早期表现为反复高热，复查CLL/SLL呈CR状态，但随着疾病进展出现噬血细胞综合征表现，淋巴结活检证实存在ALTCL，且是ALK^+^高增殖活性（Ki-67 80％阳性）的具有细胞毒特性（EMA^+^，TIA-1^+^和颗粒酶B^+^）的T细胞淋巴瘤，肿瘤部位IgH和TCRβ基因的单克隆重排均阳性，考虑ALTCL的IgH重排来源于CLL/SLL。Nai等[20]介绍了一例出现脾大伴血小板减少的CLL患者，脾切除后病理证实为ALTCL，被认为是一种不常见的RS。有研究报道一例CLL/SLL患者，出现ALK阴性ALTCL和HL[21]。遗传学分析显示其等位基因的IgH基因重排，ALTCL、HL与CLL携带相同的体细胞突变，证实CLL/SLL细胞具有转化为ALTCL和HL形态淋巴瘤可能。Liu等[22]报道，一例有8年CLL病史的患者发展为ALK阳性间变大细胞淋巴瘤（ALCL），且淋巴结及骨髓活检均证实同时存在CLL和ALCL两种病理形态，分子学检测示IgH重排和TCRγ、TCRβ基因的单克隆重排均可检测到，证实其为复合型肿瘤。

ALK^+^ALTCL是外周T细胞淋巴瘤中预后较好的类型，本文病例中ALK在细胞质及细胞核中均有表达，但应用西达本胺联合化疗后患者疾病持续进展，很快死亡，呈现出与初始ALCL不一样的病程特点，也侧面反映该患者的ALK^+^ALTCL可能是由CLL/SLL转化的高侵袭性淋巴瘤，是RS的一种变体，目前为国内首次报道。
